# A simple and effective method for simulating nested exchangeable correlated binary data for longitudinal cluster randomised trials

**DOI:** 10.1186/s12874-024-02285-4

**Published:** 2024-08-08

**Authors:** Rhys A. Bowden, Jessica Kasza, Andrew B. Forbes

**Affiliations:** School of Public Health and Preventive Medicine, Monash University, Melbourne, Australia

**Keywords:** Simulation, Correlated binary random variables, Nested exchangeable correlation structure, Longitudinal cluster randomised trials, Block exchangeable correlation structure, Hierarchical models, Multi-level models

## Abstract

**Background:**

Simulation is an important tool for assessing the performance of statistical methods for the analysis of data and for the planning of studies. While methods are available for the simulation of correlated binary random variables, all have significant practical limitations for simulating outcomes from longitudinal cluster randomised trial designs, such as the cluster randomised crossover and the stepped wedge trial designs. For these trial designs as the number of observations in each cluster increases these methods either become computationally infeasible or their range of allowable correlations rapidly shrinks to zero.

**Methods:**

In this paper we present a simple method for simulating binary random variables with a specified vector of prevalences and correlation matrix. This method allows for the outcome prevalence to change due to treatment or over time, and for a ‘nested exchangeable’ correlation structure, in which observations in the same cluster are more highly correlated if they are measured in the same time period than in different time periods, and where different individuals are measured in each time period. This means that our method is also applicable to more general hierarchical clustered data contexts, such as students within classrooms within schools. The method is demonstrated by simulating 1000 datasets with parameters matching those derived from data from a cluster randomised crossover trial assessing two variants of stress ulcer prophylaxis.

**Results:**

Our method is orders of magnitude faster than the most well known general simulation method while also allowing a much wider range of correlations than alternative methods. An implementation of our method is available in an R package NestBin.

**Conclusions:**

This simulation method is the first to allow for practical and efficient simulation of large datasets of binary outcomes with the commonly used nested exchangeable correlation structure. This will allow for much more effective testing of designs and inference methods for longitudinal cluster randomised trials with binary outcomes.

**Supplementary Information:**

The online version contains supplementary material available at 10.1186/s12874-024-02285-4.

## Background

Binary data arises in naturally in many fields. There are a wide variety of models and statistical methods for such data, and it is important to be able to evaluate the appropriateness and validity of these methods by numerical simulation. These simulation studies are vitally important tools used in the development of statistical methodology [1]. The simulation of correlated binary data is of particular importance in the investigation of methods for the planning and analysis of cluster randomised trials. These are trials where clusters of individuals are randomised to a particular treatment, and longitudinal cluster randomised designs extend these designs to randomization of clusters to a sequence of treatments over disjoint time periods. Binary outcome data are common in such trials: for example, a systematic review of stepped wedge cluster randomised trials found that 34 out of the 60 identified trials had binary primary outcomes [2].

There are a number of methods available to simulate correlated binary data. For some models where a full joint distribution of the observations is assumed (for example, the distribution arising from a logistic regression model) the model often suggests a straightforward way to simulate the data. However, in many other cases, the researcher is concerned only with the first and second moments (means and covariances of observations) of the data. Marginal models, specifying just the mean response and the covariance structure (i.e. the first two moments), have the advantage that they avoid the need to specify a full multivariate model. In such cases, although the different simulation methods may give rise to joint distributions with different higher order moments, this is not an issue when interest lies solely in these first and second moments. However, despite the freedom from concern regarding specification of higher order moments, there are still major obstacles to effective simulation.

The first major obstacle is computation speed: several currently-available methods are impractically slow when simulating data from large clusters [3–6]. The second obstacle is that several currently available methods only allow simulation for a restrictive range of intracluster correlations [7, 8]. Although not all methods suffer from both of these drawbacks there are several frequent and important situations in which researchers wish to simulate data from large clusters for a wide range of possible intracluster correlations. One such situation is in sample size and power calculations for longitudinal cluster randomised trials, such as the PEPTIC trial [9] or the Riverbank filtration stepped wedge trial [10]. The PEPTIC trial compared two different drugs for the prevention of stress ulcers in patients in intensive care units. This trial randomised 50 entire intensive care units in several countries to two different treatment sequences. Intensive care units either treated all of their eligible patients with the standard drug for six months before switching to the novel treatment for a second six month period, or vice-versa. This ‘cluster crossover’ trial randomised intensive care units, with an average of 310 patients in each unit in each of the two study periods, with different patients in each unit and study period.

To properly validate sample size calculations for the PEPTIC trial, and other such longitudinal cluster randomised crossover trials with large cluster sizes, researchers may often wish to simulate data from such a trial for a range of intracluster correlations, and for different within-cluster correlation structures. Initially, the most commonly considered correlation structure for longitudinal cluster randomised trials was the *exchangeable* one: any pair of measurements in the same cluster had the same correlation, regardless of which time periods they were measured in [11]. However, it has more recently become recognised that it is often necessary to allow for different intra-cluster correlations depending on whether measurements come from the same measurement period or different measurement periods [12–14], and our method is targeted at this case.

The paper is structured as follows: The remainder of this Background section formalises the prevalence and correlation structures we are interested in simulating then describes existing simulation methods and their limitations. The Methods section presents our simulation model and method. In the Results section we compare its computational efficiency and correlation bounds with existing methods, then present an example of simulating data similar to the PEPTIC trial. We close with the Conclusions section, including a discussion of the method’s salient features and extensions.

### Problem formulation

Our objective is to simulate correlated binary data with two nested levels of clustering and a nested exchangeable correlation structure [15]. That is, each observation $W_{i,j,k}\in \{0,1\}$ belongs to cluster $i \in \{1,\ldots ,C\}$, and each cluster *i* is partitioned into subclusters $j=1,\ldots ,T_i$, (these could correspond to cluster-periods in longitudinal cluster randomised trials) each of which contains observations $k=1,\ldots ,N_{i,j}$. In this work, individuals are only measured in one time period, often referred to as cross-sectional sampling. Although we have described above a setting for a longitudinal cluster randomised trial, there are many other examples of hierarchical clustering of observations: for instance, subclusters nested within clusters might correspond to classes nested within schools or medical clinics nested within geographical areas [16].

Since observations in different clusters are independent, this problem reduces to determining a method to efficiently simulate data from a single cluster (and then repeating that sampling with the same or different parameters, potentially a large number of times). Because we are only considering one cluster at a time for notational simplicity we omit the subscript *i* from $W_{i,j,k}$, $T_i$ and $N_{i,j}$. Further, since only one cluster is considered at a time, the clusters are able to have differing parameters such as correlations and prevalences.

Given the correlations $\rho _C$, $\rho _{CT}$, the number of subclusters *T*, the number of observations in the *j*th subcluster $N_{j}$ and a vector of prevalences $\varvec{\pi }=(\pi _1,\ldots ,\pi _T)$ we wish to generate binary random variables $W_{j,k}\in {0,1}$ such that

1 $$\begin{aligned} \text {Cor}(W_{j_1,k_1},W_{j_2,k_2})&=\rho _C, \end{aligned}$$

2 $$\begin{aligned} \text {Cor}(W_{j,k_3},W_{j,k_4})&= \rho _{CT} \ge \rho _C,\ \text {and}\end{aligned}$$

3 $$\begin{aligned} \text {Pr}(W_{j,k}=1)&=\pi _j, \end{aligned}$$

for all $j,j_1,j_2\in \{1,\ldots ,T\}$; $k_1\in \{1,\ldots ,N_{j_1}\}$; $k_2\in \{1,\ldots ,N_{j_2}\}$; $k,k_3,k_4\in \{1,\ldots ,N_j\}$ with $j_1 \ne j_2$ and $k_3 \ne k_4$. Here $\rho _C$ is referred to as the *between subcluster* correlation and $\rho _{CT}$ is known as the *within subcluster* correlation. We have included the typical constraint that $\rho _C\le \rho _{CT}$. Block exchangeable correlation structures where $\rho _C>\rho _{CT}$ are generally not considered in cluster randomised trials, due to the lack of a realistic justification for there to be greater correlation between pairs of observations in different periods rather than those in the same period. Similarly, negative correlations are not usually considered for even moderately large clusters, due to the constraint that *n* mutually correlated binary variables must have correlations that are greater than $-\frac{1}{n-1}$.

For example, if there are $T=2$ periods and $N_{1}=N_{2}=3$ observations per cluster-period cell in a given cluster then the correlation matrix *R* for the observations from that cluster will be:

It is important to allow for a separate prevalence in each cluster-period because models for outcome data from longitudinal cluster randomised trials must allow for different treatment conditions in different cluster-periods, and those treatments are often intended to have an effect on the prevalence. Further, it is common to allow for changes in prevalence due to the time period. A common model for the prevalence in cluster *i*, period *j* is

4 $$\begin{aligned} \pi _{i,j} = \beta _j + D_{i,j} \theta \end{aligned}$$

where $\beta _j$ and $\theta$ are time effects and treatment effect respectively, and $D_{i,j}$ is the treatment indicator, with $D_{i,j}=1$ if the intervention is applied in period *j* of cluster *i*, and 0 otherwise.

### Existing simulation methods

Consider the more general task of simulating *d* dependent binary variables, $\textbf{W}=(W_1,\ldots ,W_d) \in \{0,1\}^d$ so that they have the prespecified prevalences (means) $\varvec{\pi } = (\pi _1,\ldots ,\pi _d)$ and prespecified correlation matrix *R*. In the context of the formulation given above, typically $d=\sum _{j=1}^T N_j$. There have been many methods developed to address this problem, with differing conditions on $\varvec{\pi }$ and *R* and differing levels of complexity and computational efficiency (see Farrell and Rogers-Stewart [17] for a survey).

Perhaps the most straightforward simulation approach is to determine the full joint distribution of $\textbf{W}=(W_1,\ldots ,W_d)$ (this may require specifying the probability of each of the $2^{d}$ outcomes for $\textbf{W}$) and then sample from this distribution. Several authors propose such methods [3–5]. However, computationally this approach scales very poorly as *d* increases and is infeasible when $d=100$ or more, as frequently occurs in longitudinal cluster randomised trials, like the PEPTIC [9] trial mentioned above.

Other methods are more computationally efficient, but place restrictions on the form of $\varvec{\pi }$ and *R*. For instance, Lunn and Davies [18] only allow for generating *stationary* data, that is, one common prevalence $\pi _1=\ldots =\pi _d$ and an exchangeable or autoregressive(1) correlation structure. An exchangeable correlation matrix has all off-diagonal elements identical, and the correlation matrix for an autoregressive(1) process has the (*a*, *b*) entry given by $R(a,b)=\rho _0^{|a-b|}$ for some $\rho _0\in (-1,1)$. Oman and Zucker [19] extend Lunn and Davies [18] to allow for general $\varvec{\pi }$ and the MA(1) correlation structure. An MA(1) correlation structure (that of a moving average process of order 1) corresponds to a correlation matrix with (*a*, *b*) entry 1 if $a=b$, some correlation value $\rho _1$ if $|a-b|=1$ and 0 otherwise [19]. Lee [20] also allows for a general $\varvec{\pi }$, but their correlation structure has only one correlation parameter $\rho$. As far as we are aware, none of the methods with a specific form of correlation matrix (such as an exchangeable correlation matrix, or a Toeplitz correlation matrix arising from an autoregressive correlation structure, for example) allow for a nested exchangeable correlation structure.

There are other methods that allow a more general form of prevalences and correlation matrix. Emrich and Piedmonte [6] describe a method involving simulation of data from a carefully constructed multivariate normal distribution, and then dichotomising the samples to obtain binary variables. The thresholds for dichotomisation are determined by the prevalences of the individual binary random variables, and then the covariance matrix for the multivariate normal distribution is numerically determined to match the required correlations of the binary variables. The main weakness of this method is the computational time required; first to calculate the required covariance matrix of the multivariate normal distribution and then to sample a (possibly high dimensional) multivariate normal random variable for each multivariate binary sample. This can be impractical for sampling longitudinal cluster randomised trials, such as PEPTIC, with 310 observations in each cluster-period.

There have been other methods developed to simulate binary data more efficiently than Emrich and Piedmonte [6] for cases where the dimension of the required data, *d*, is large. Often such methods generate simulated data sequentially from random variables whose marginal distributions conditioned on those already sampled are easy to calculate. Two examples are the methods of Jiang et al. [7] and Qaqish [8]. In practice these methods place quite stringent restrictions on the set of allowable covariances or correlations (we say that a pair of a prevalence vector and correlation matrix is *allowable* for a simulation method if that method can simulate binary random vectors with those prevalences and correlations), and lead to severe restrictions for the nested exchangeable correlation structure that we are considering. Furthermore, analytically evaluating the possible range of correlations can be very difficult, even in cases with a very small dimension. Even for $d=3$, the range of mathematically possible correlation matrices is not easy to determine [21]. Chaganty and Joe [21] also compare the range of correlations possible with the methods of Emrich and Piedmonte [6] and Qaqish [8] for a few specific scenarios with $d=3$. For some of these scenarios one of the two methods comes very close to allowing the maximal theoretical correlation derived by Chaganty and Joe [21]. This is potentially misleading because the range of allowable correlations shrinks dramatically as *d* increases (as we later show in Fig. 3). Preisser and Qaqish [22] do a more extensive comparison of the allowable correlations for these two methods over a variety of scenarios. They show that in some cases the method of Qaqish [8] attains the maximum mathematically possible allowable correlations, but in others the method of Emrich and Piedmonte [6] has a wider range of allowable correlations. Most of their examples considered have $d\le 6$, but one example considers *d* ranging from 4 to 220, and shows that higher *d* can have a substantial deleterious effect on the maximum allowable positive correlation. In the Results section we give an empirical comparison of the ranges of allowable correlations for the methods of Jiang et al. [7] and Qaqish [8] when applied to the nested exchangeable case, with $d=100$. We show that the range of allowable correlations is much smaller than that of the method we propose. Further, the range of allowable correlations continues to shrink as *d* increases, to the point that neither Jiang et al.’s [7] nor Qaqish’s [8] method are practical for simulating data from typical longitudinal cluster randomised trials.

In the Methods section we present a method that is tailored to nested exchangeable data such as that from longitudinal cluster randomised trials. In the Results section we show that our method has a range of allowable correlations that is often an order of magnitude larger than that given by Jiang et al. [7] or Qaqish [8], while also executing much more quickly than the method of Emrich and Piedmonte [6].

## Methods

### Structure of simulation model

In our approach we model $W_{j,k}$ as a mixture of binary variables, one for each level of nesting (i.e. clustering):

5 $$\begin{aligned} W_{j,k} = \left\{ \begin{array}{l} X_{j,k}\ \text {with probability}\ m_j\\ Y_{j}\ \text {with probability}\ \widetilde{m}_j\\ Z\ \text {with probability}\ \overline{m}_j \end{array}\right. \end{aligned}$$

such that

6 $$\begin{aligned} m_j+\widetilde{m}_j+\overline{m}_j=1 \end{aligned}$$

for all $j=1,\ldots ,T$; $k=1,\ldots ,N_j$ and $X_{j,k},Y_{j},Z$ are independent Bernoulli random variables with prevalences

7 $$\begin{aligned} \text {Pr}(X_{j,k} = 1)&= x_j\end{aligned}$$

8 $$\begin{aligned} \text {Pr}(Y_{j} = 1)&= y_j\end{aligned}$$

9 $$\begin{aligned} \text {Pr}(Z = 1)&= z. \end{aligned}$$

Even though higher order moments and the full joint distribution are not specified in the problem formulation (as described in the Background section above), any sampling method implicitly or explicitly gives a model for the whole joint distribution. We have chosen a model that is as parsimonious as possible, while still allowing for satisfying the requirements given in the Problem formulation section, and also respecting the symmetries of the correlation structure.

This model results in the between subcluster correlation for subclusters $j_1,j_2$ with $j_1\ne j_2$ being

10 $$\begin{aligned} \rho _{C}&= \frac{\text {Cov}(W_{j_1,k_1},W_{j_2,k_2})}{\sqrt{\text {Var}(W_{j_1,k_1})\text {Var}(W_{j_2,k_2})}}\end{aligned}$$

11 $$\begin{aligned} &= \frac{\text {Pr}(W_{j_1,k_1}=1,W_{j_2,k_2}=1)-\text {Pr}(W_{j_1,k_1}=1)\text {Pr}(W_{j_2,k_2}=1)}{\sqrt{\pi _{j_1}(1-\pi _{j_1})\pi _{j_2}(1-\pi _{j_2})}}\end{aligned}$$

12 $$\begin{aligned} &= \frac{\overline{m}_{j_1}\overline{m}_{j_2}z(1-z)}{\sqrt{\pi _{j_1}(1-\pi _{j_1})\pi _{j_2}(1-\pi _{j_2})}}. \end{aligned}$$

Equation (12) follows from Eq. (11) because the only case in which $W_{j_1,k_1}$ and $W_{j_2,k_2}$ are not independent is when they are both equal to *Z* which happens with probability $\overline{m}_{j_1}\overline{m}_{j_2}$. In that case, the covariance will be $\text {Var}(Z)=z(1-z)$.

The within subcluster correlation for cluster *j* and observations $k_1,k_2$ with $k_1 \ne k_2$ are

13 $$\begin{aligned} \rho _{CT}&= \frac{\text {Cov}(W_{j,k_1},W_{j,k_2})}{\sqrt{\text {Var}(W_{j,k_1})\text {Var}(W_{j,k_2})}}\end{aligned}$$

14 $$\begin{aligned} &=\frac{\widetilde{m}_j^2y_j(1-y_j)+\overline{m}_{j}^2z(1-z)}{\pi _{j}(1-\pi _{j})} \end{aligned}$$

using similar reasoning to that given above.

Hence the problem reduces to choosing $m_j,\widetilde{m}_j,\overline{m}_j,x_j,y_j,z$ to satisfy the system of Eqs. (6), (12), (14) and the required prevalences

15 $$\begin{aligned} \pi _j = m_j x_j + \widetilde{m}_j y_j + \overline{m}_j z \end{aligned}$$

while also requiring that

16 $$\begin{aligned} 0\le m_j,\widetilde{m}_j,\overline{m}_j,x_j,y_j,z \le 1. \end{aligned}$$

There can be more equations than variables ($(T^2+5T)/2$ equations and $5T+1$ variables) so there need not be a feasible solution, however our model is chosen in such a way that this does not cause an issue, the only barrier is whether or not (16) can also be satisfied.

### Parameter values

Here we provide values of the simulation parameters $x_j,y_j,z,m_j,\widetilde{m}_j,\overline{m}_j$ that simultaneously satisfy the required Eqs. (6), (12), (14), (15), and (16).

First, let $r= 2\sqrt{\rho _C}/(1+2\rho _C-\rho _{CT})$. Then if $r>1$ the values of $\rho _C$ and $\rho _{CT}$ do not allow for simulation using our algorithm. Otherwise, let $q_{W_j} = \sqrt{\pi _j/(1-\pi _j)}$, then set

17 $$\begin{aligned} q_{Z,L} = \underset{j}{\max} q_{W_j} \frac{1-\sqrt{1-r^2}}{r};\quad q_{Z,U} = \underset{j}{\min} q_{W_j} \frac{1+\sqrt{1-r^2}}{r}. \end{aligned}$$

Now if $q_{Z,L}>q_{Z,U}$ then $\varvec{\pi }$ is not compatible with $(\rho _{CT},\rho _C)$. Otherwise, choose

18 $$\begin{aligned} q_Z=(q_{Z,L}+q_{Z,U})/2;\quad \ \text {and}\ \quad q_{Y_j} = \sqrt{\frac{q_{W_j}-\sqrt{\rho _C}q_Z}{1/q_{W_j}-\sqrt{\rho _C}/q_Z}} \end{aligned}$$

for $$j=1,\ldots ,T$$. Then

19 $$\begin{aligned} z = q_Z^2/\left(1+q_Z^2\right) \quad \ \text {and}\ \quad y_j = q_{Y_j}^2/ \left(1+q_{Y_j}^2\right) \end{aligned}$$

and

20 $$\begin{aligned} \overline{m}_{j} = \sqrt{\frac{\rho _C \pi _{j}(1-\pi _{j})}{z(1-z)}},\qquad \widetilde{m}_j&= \sqrt{\frac{(\rho _{CT}- \rho _C)\pi _{j}(1-\pi _{j})}{y_j(1-y_j)}},\qquad m_j = 1-\widetilde{m}_j - \overline{m}_j, \end{aligned}$$

21 $$\begin{aligned} x_j&= \frac{\pi _j - \widetilde{m} y - \overline{m} z}{m_j}, \end{aligned}$$

for $j=1,\ldots ,T$. The data can now be simulated according to Eqs. (5)-(9). More details on how these parameters were determined are given in Appendix 1 [see Additional File 1].

### Ranges of allowable prevalences and correlation parameters $(\varvec{\pi },\rho _{CT},\rho _C)$

If $r = \frac{2\sqrt{\rho _C}}{1+2\rho _C-\rho _{CT}}>1$ then it is not possible to simulate any data with the pair $(\rho _{CT},\rho _C)$ using this method. The constraint $r\le 1$ is equivalent to at least one of

1. $$\rho _{CT}<1/2$$, or
2. $$\rho _C \notin [\frac{\rho _{CT}-\sqrt{2\rho _{CT}-1}}{2},\frac{\rho _{CT}+\sqrt{2\rho _{CT}-1}}{2}]$$, if $$\rho _{CT}\ge 1/2$$

being true. The situation where $\rho _{CT}>\frac{1}{2}$ is very uncommon in cluster randomised trials [23–25] particularly for binary data when the ICC is estimated on the natural scale (as it is here). For instance, [26] lists a summary of ICCs from a broad variety of studies and reports that 99% of unadjusted ICCs were below 0.272. This means that the constraint of $r\le 1$ is very unlikely to be violated.

If $r\le 1$, the other way in which this method might fail is if $q_{Z,L}>q_{Z,U}$. In that case, $\varvec{\pi }$ is not compatible with $(\rho _{CT},\rho _C)$. This is primarily driven by the interval $(\min _j \pi _j,\max _j \pi _j)$ being too wide, or the correlations $\rho _{CT},\rho _C$ causing *r* to be too close to 1.

## Results

### Comparison of methods

**Fig. 1:**
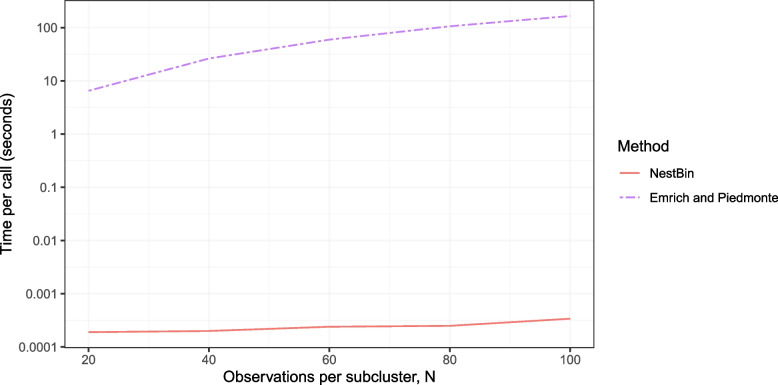
The time in seconds to simulate binary data with 2 clusters and 8 subclusters in each cluster vs the number of observations in each cluster *N*. Two methods are compared: the method of Emrich and Piedmonte (dashed line), implemented for R in the package rmvbin and our nested exchangeable binary method (NestBin, solid line)

We compare our method to the most commonly used alternatives: the methods of Emrich and Piedmonte [6], Qaqish [8], and Jiang et al. [7] to show that it does not suffer from the same problems that make those methods impractical in settings such as longitudinal cluster randomised trials. We first compare the run time of our method against that of Emrich and Piedmonte [6] in Fig. 1. Methods were run on a desktop PC in R version 4.4.0 using the 0.9.21 version of the bindata package for the Emrich and Piedmonte method (another R package implementing this method, mvtBinaryEP, performed similarly). Even with only 8 subclusters our method is $10^5$ to $10^6$ times faster than Emrich and Piedmonte’s method depending on the number of observations per subcluster *N*. In practical terms, despite our method’s vastly superior speed, Emrich and Piedmonte’s method could still be manageable with a small number of total observations per cluster *TN*. However, as *TN* increases, that method slows down dramatically and becomes computationally infeasible. This is due to the requirement of sampling a multivariate normal random variable of dimension *TN*. In contrast, our method requires sampling only $TN+T+1$ *independent* binary random variables and so is much faster.

To evaluate the usefulness of our proposed method, we compare the range of allowable values of $\rho _{CT}$ (the within cluster-period ICC) using our method with the range of allowable values of $\rho _{CT}$ for the method of Jiang et al. [7] and the method of Qaqish [8]. We fix $N_1=\cdots =N_T=N$ for these comparisons, and simulate periods alternating between a prevalence of $\pi _{\text {min}}$ and a prevalence of $\pi _{\text {max}}$, where $\pi _{\text {max}}>\pi _{\text {min}}$. We refer to $\pi _{\text {max}}-\pi _{\text {min}}$ as the *range*. For Qaqish’s method we use the algorithm given in the paper [8] to evaluate whether a given set of prevalences and correlations are able to be simulated. However, the highest allowable correlation depends on the order in which the variables are sampled. Testing any given permutation of the variables requires inverting a square matrix whose dimensions are equal to the number of observations *TN*, so it is not feasible to test every given one of the (*TN*)! permutations. Qaqish [8] suggests trying several random permutations, however we found that the choice of permutation made negligible difference in practice. For Jiang et al.’s method we use their supplied R package to test whether a given set of prevalences and correlations are able to be simulated by implementing the algorithm given in their paper to do so.

**Fig. 2:**
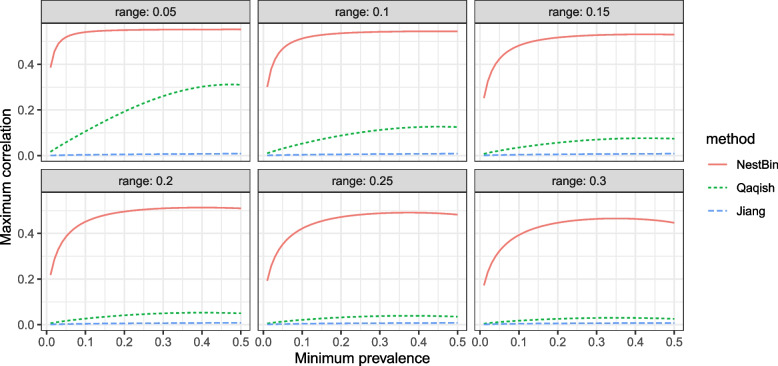
The maximum allowable values of the within subcluster correlation $\rho _{CT}$ for given ranges of the prevalences, for each method with $T=10$ and $N=20$. The solid red line is our method NestBin, the short-dashed green line is the method of Qaqish, and the long-dashed blue line is the method of Jiang et al. The x axis displays the minimum prevalence and each subplot corresponds to a fixed difference between maximum and minimum prevalence. For instance, a minimum prevalence of 0.1 and range of 0.35 means that prevalences would be in the interval [0.1,0.45]. With a wider range of prevalences, the maximum allowable $\rho _{CT}$ is decreased. For this figure, the cluster level correlation is $\rho _C = 0.8\rho _{CT}$

Figure 2 shows the maximum allowable values of $\rho _{CT}$ plotted against the minimum prevalence, and the difference between the maximum and minimum prevalence, labelled as *range* on the plot (since the combination of maximum and minimum prevalence defines which correlations are allowable). The allowable correlations from Jiang’s method are generally less than or equal to 0.04, whereas those of our method are always much higher, reaching up to around 0.55 when the interval of required prevalences is small or the minimum prevalence is close to 1/2. Qaqish’s method allows for a reasonable maximum correlation of over 0.3 when the range of prevalences is narrow and centred around 1/2, but rapidly gets worse for wider ranges of prevalences or low values of minimum prevalence.

**Fig. 3:**
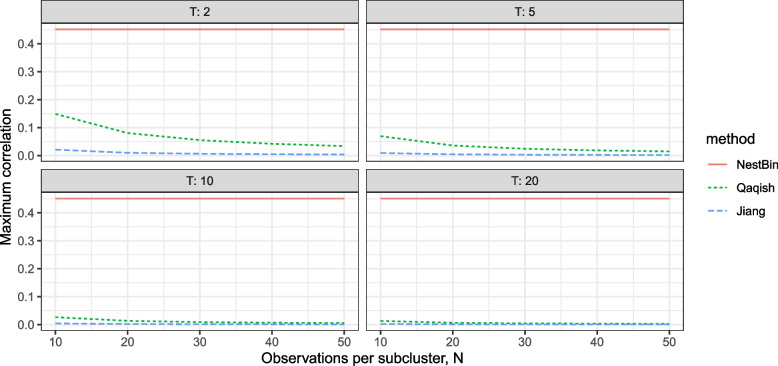
The maximum allowable values of the within subcluster correlation $\rho _{CT}$ for prevalences ranging from 0.1 to 0.3, for each method over varying values of *T* and *N*. The solid red line is our method NestBin, the short-dashed green line is the method of Qaqish, and the long-dashed blue line is the method of Jiang et al. As the number of observations increases, the maximum allowable $\rho _{CT}$ decreases for the Qaqish and Jiang et al. methods, but it is independent of *N* and *T* for our method. For this figure, the cluster level correlation is $\rho _C = 0.8\rho _{CT}$

Further, the results in Fig. 2 are for only 200 observations per cluster, totalled across all subclusters: this represents very few observations for a longitudinal cluster randomised trial, and far less than the 620 in the PEPTIC trial. Figure 3 presents the maximum allowable values of the within subcluster correlation $\rho _{\text {CT}}$ for prevalences in [0.1,0.3] for each method over varying values of number of subclusters *T* and observations per subcluster *N*. Importantly, the number of observations has no effect on the range of feasible correlations for our method, whereas increasing the number of observations reduces the range of feasible correlations for both the Jiang et al. [7] and Qaqish [8] methods. As *N* and *T* increase, even modest values of within-subcluster correlation become unattainable. Thus, those methods are typically not practical for simulating data with the desired structure and large cluster sizes.

**Fig. 4:**
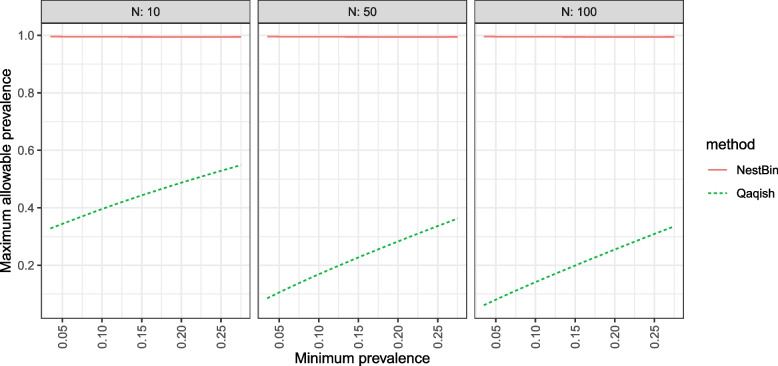
The maximum allowable prevalence $\pi _{\text {max}}$ for a given minimum prevalence $\pi _{\text {min}}$; with $\rho _{CT}$ determined by $\log (\rho _{CT}) = 0.61\log (\pi _{\text {min}}) -2.1$, for $T=6$ periods and a cluster autocorrelation of $\rho _C/\rho _{CT} =0.8$. The solid red line is our method NestBin, the short-dashed green line is the method of Qaqish, and the method of Jiang does not appear here because it was not able to simulate any of these configurations

We can also take a different perspective on which combinations of correlations and prevalences are possible to simulate: rather than find the highest allowable correlation for a given range of prevalences $[\pi _{\text {min}},\pi _{\text {max}}]$ we can ask, what is the highest allowable prevalence $\pi _{\text {max}}$ for a given $\rho _{CT},\rho _C$ and $\pi _{\text {min}}$? This is of practical importance, because a treatment effect would typically involve a change in prevalence (time effects might also change the prevalence for example, see Eq. 4), so the highest positive treatment effect that could be simulated would be less than or equal to $\pi _{\text {max}}-\pi _{\text {min}}$. We show the highest allowable values for $\pi _{\text {max}}$ over a range of ICCs and prevalences derived from the empirical observations of Gulliford et al. [23]. That work draws from two databases of clinical records of chronic diseases and shows that higher ICCs were associated with higher prevalences, where clusters were treatment practices in the United Kingdom. For the larger of two databases, the General Practice Research Database, there were 188 ICCs with a median prevalence of 13.1% (interquartile range 3.5% to 28.4%) and median ICC 0.051 (IQR 0.011 to 0.094). They found a linear association of log ICC with log prevalence with a regression coefficient 0.61 and intercept of -2.10. We use ICCs corresponding to their interquartile range (0.011 to 0.094) and determine $\pi _{\text {min}}$ using the relationship they estimated, $\log (\rho _{CT}) = 0.61\log (\pi _{\text {min}}) -2.1$, then plot the highest allowable values of $\pi _{\text {max}}$ for each of three methods: our method and those of Jiang et al. [7] and Qaqish [8]. The results are presented in Fig. 4. We can see that our method allows a much broader range of possible intervention effects ($\pi _{\text {max}}-\pi _{\text {min}}$) than those of Qaqish and Jiang. The method of Qaqish performs reasonably for small *N*, but again the performance deteriorates as *N* increases. The method of Jiang cannot simulate these correlations and prevalences, even with $\pi _{\text {max}}=\pi _{\text {min}}$, and as such does not appear in the figure.

### Demonstration of method

We demonstrate our method using an example based on the design of the PEPTIC trial, a cluster crossover trial comparing two approaches to stress ulcer prophylaxis in mechanically ventilated adults [27]. In this case, the study protocol document assumed 50 clusters, a baseline prevalence of 0.15 and an additive treatment effect of -0.024, leading to prevalences of $(\pi _1,\pi _2) = (0.15,0.126)$. The average number of patients per cluster-period was assumed to be $N=310$, with a within-cluster-within-period correlation of $\rho _{CT} = 0.035$ and a within-cluster-between-period correlation of $\rho _C=0.025$. We use our method to sample with these parameters, but rather than sample 50 (independent) clusters as in PEPTIC, we instead sample 1000 clusters and use this large sample to confirm that we are obtaining the desired prevalences and correlations when using our sampling procedure. For the purposes of this demonstration, we are not modelling additional time effects, so for simplicity (and without loss of generality) we reorder the cluster-periods so that each cluster has a higher prevalence in the first time period, and a lower prevalence in the second time period.

**Fig. 5:**
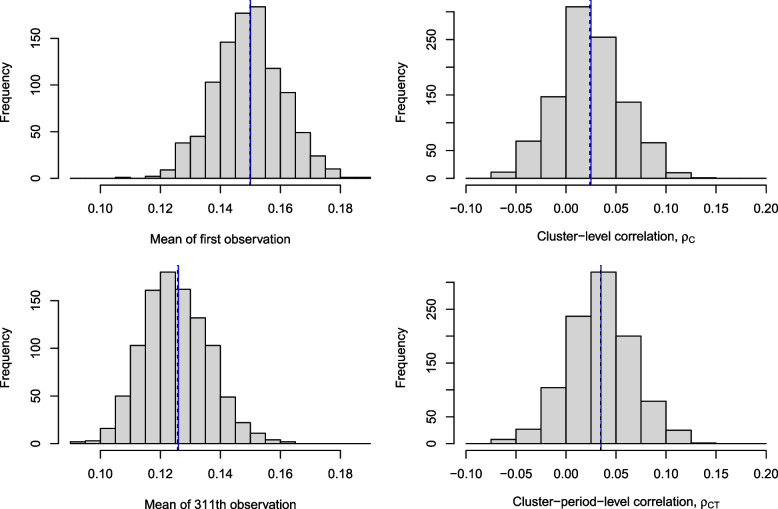
Histograms of 1000 mean and correlation estimates for each of (1) the first observation in the first time period of the cluser (top left), (2) the first observation in the second time period of the cluster (bottom left), (3) the correlation between the first observations from each of the first two periods in the cluster (top right), and (4) the correlation of the first and second observations in the cluster. The vertical black line is the mean of the estimates, and the vertical blue line is the desired value (overlaying the black line in some cases). The desired values are $\pi _1=0.15$, $\pi _2=0.126$, $\rho _C=0.025$ and $\rho _{CT}=0.035$ respectively

Each cluster is independently sampled, so we can then take the mean of the 1000 samples of the first observation in each cluster and check that this mean is approximately 0.15 as desired. We can do the same for the first observation in the second period of each cluster to check that their mean is approximately 0.126. We can also estimate the correlation between the first and second observation in the each cluster (the two observations in the first period), using the sample correlation. Finally, we can estimate the correlation between the first observation in the first period and the first observation in the second period of each cluster. This process gives us one estimate for each prevalence and correlation parameter from one random sample of 1000 clusters. We repeat this process 1000 times to get 1000 independent estimates, and plot the results in Fig. 5. As expected, the prevalences and correlations are centred around the desired values.

## Conclusions

We have developed a highly efficient and effectives method for simulating binary random variables with given prevalences and a nested exchangeable correlation structure. This method is important in particular for the study of longitudinal cluster randomised trials with either a large number of subjects per cluster, in which case our method has extreme computational advantages, or high within cluster correlations, which our method has a greater capacity to handle. Typical values of within-cluster-period correlation $\rho _{CT}$ would be expected to be far below 0.5 in most settings, with the possible exception being process outcomes [23]. Efficient simulation is required in these cases to not only assess the performance of new methods for analysis of data, but also in sample size and power calculations, where researchers often conduct simulation studies to validate analytical formulae. However, this method is not solely applicable to longitudinal cluster randomised trials, it is useful in any case of binary outcomes where hierarchical clustering leads to a nested exchangeable correlation structure. Such examples include students nested within classrooms within schools, individuals nested within households within communities, or patients nested within doctors within clinics.

The main limitation of the approach presented in this paper is that it is as yet applicable only to exchangeable and nested exchangeable correlation structures, although those are commonly assumed in the design and analysis of cluster randomised trials [2, 25]. Other correlation structures such as discrete time decay [28] are still being investigated for modelling longitudinal cluster randomised trials with binary outcomes. Also, in longitudinal trials like PEPTIC, patients can be recruited and observations taken continuously over time, in which case it could make more sense to model correlations as varying continuously in time rather than discretised into periods, see for example Grantham et al. [29]. However, this limitation is also a strength of the method: because the correlation structure is specified it allows for a more targeted simulation method that is relatively simple, more computationally efficient and allows for a higher range of correlations than more general methods. We believe that the methods with general correlation structures (Jiang et al. [7] and Qaqish [8]) struggle with simulating this correlation structure because both methods are based on generating the data serially: each observation is sampled individually, with a distribution conditional on the observations before it. This is consistent with the idea of the data being serial observations from a time series, and so does not exploit the symmetries inherent in data with a nested exchangeable correlation structure.

We have implemented the method presented in this paper in an R package called NestBin available at https://github.com/rhysbowden/NestBin .

### Supplementary Information

Supplementary Material 1.

## Data Availability

The R package NestBin is available at: https://github.com/rhysbowden/NestBin Scripts to generate the plots in this paper are available at: https://github.com/rhysbowden/NestBin-examples Results and plots were generated using R version 4.4.0, CorBin version 1.0.0 and NestBin version 1.0.0.

## Abbreviation

ICC: Intra-cluster correlation coefficient
