# Supplementary material for: A simple and effective method for simulating nested exchangeable correlated binary data for longitudinal cluster randomised trials
Source: BMC Med Res Methodol. 2024 Aug 8;24:174. doi: 10.1186/s12874-024-02285-4 (PMC11308151; doi:10.1186/s12874-024-02285-4)
Supplement: Supplementary file 1 — Supplementary Material 1. [file 12874_2024_2285_MOESM1_ESM.pdf]

RESEARCH

A simple and effective method for simulating nested exchangeable correlated binary data for longitudinal cluster randomised trials: Appendix 1

Rhys A. Bowden*, Jessica Kasza and Andrew B. Forbes

*Correspondence:
rhys.bowden@monash.edu
School of Public Health and Preventive Medicine, Monash University, Melbourne, Australia
Full list of author information is available at the end of the article

Appendix 1: Method for finding model parameters from constraints

We start with Equation (12) and rewrite it as

$$\frac{\rho_C}{z(1-z)} = \frac{\overline{m}_{j_1}}{\sqrt{\pi_{j_1}(1-\pi_{j_1})}} \cdot \frac{\overline{m}_{j_2}}{\sqrt{\pi_{j_2}(1-\pi_{j_2})}} \tag{22}$$

so if we choose $\overline{m}_j$ and z such that

$$\overline{m}_j = \sqrt{\frac{\rho_C \pi_j(1-\pi_j)}{z(1-z)}}, \tag{23}$$

then Equation (12) will be satisfied for all $j = 1, \ldots, T$. We then substitute Equation (23) into Equation (14) to get

$$\rho_{CT}\pi_j(1-\pi_j) = \widetilde{m}_j^2 y_j(1-y_j) + \frac{\rho_C \pi_j(1-\pi_j)}{z(1-z)} z(1-z) \tag{24}$$

$$\therefore \quad \pi_j(1-\pi_j)(\rho_{CT} - \rho_C) = \widetilde{m}_j^2 y_j(1-y_j) \tag{25}$$

$$\therefore \quad \widetilde{m}_j = \sqrt{\frac{(\rho_{CT}-\rho_C)\pi_j(1-\pi_j)}{y_j(1-y_j)}}. \tag{26}$$

Substituting Equations (23) and (26) into Equation (6) and rearranging gives:

$$m_j = 1 - \sqrt{\frac{(\rho_{CT}-\rho_C)\pi_j(1-\pi_j)}{y_j(1-y_j)}} - \sqrt{\frac{\rho_C \pi_j(1-\pi_j)}{z(1-z)}}, \tag{27}$$

and substituting all of these into the prevalence constraint, Equation (15), and dividing by $\sqrt{\pi_j(1-\pi_j)}$ gives

$$\frac{\pi_j}{\sqrt{\pi_j(1-\pi_j)}} = x_j\left(\frac{1}{\sqrt{\pi_j(1-\pi_j)}} - \sqrt{\frac{\rho_{CT}-\rho_C}{y_j(1-y_j)}} - \sqrt{\frac{\rho_C}{z(1-z)}}\right) + y_j\sqrt{\frac{\rho_{CT}-\rho_C}{y_j(1-y_j)}} + z\sqrt{\frac{\rho_C}{z(1-z)}}$$

$$\therefore \sqrt{\frac{\pi_j}{1-\pi_j}} - \sqrt{\frac{(\rho_{CT}-\rho_C)y_j}{1-y_j}} - \sqrt{\frac{\rho_C z}{1-z}} \tag{28}$$

$$= x_j\left(\frac{1}{\sqrt{\pi_j(1-\pi_j)}} - \sqrt{\frac{\rho_{CT}-\rho_C}{y_j(1-y_j)}} - \sqrt{\frac{\rho_C}{z(1-z)}}\right)$$

$$\therefore x_j = \frac{\sqrt{\frac{\pi_j}{1-\pi_j}} - \sqrt{\frac{(\rho_{CT}-\rho_C)y_j}{1-y_j}} - \sqrt{\frac{\rho_C z}{1-z}}}{\frac{1}{\sqrt{\pi_j(1-\pi_j)}} - \sqrt{\frac{\rho_{CT}-\rho_C}{y_j(1-y_j)}} - \sqrt{\frac{\rho_C}{z(1-z)}}}. \tag{29}$$

So now it suffices to choose $0 < y_j, z < 1$ for $j = 1, \ldots, T$ such that the quantities defined in Equations (27) and (29) are all simultaneously between 0 and 1. If this is done successfully then that solution will also guarantee $0 < \widetilde{m}_j, \overline{m}_j < 1$ for all $j = 1, \ldots, T$ due to Equation (6).

Starting with $x_j < 1$ in Equation (29) we can multiply both sides by the denominator because $m_j > 0$. This gives

$$\sqrt{\frac{\pi_j}{1-\pi_j}} - \sqrt{\frac{(\rho_{CT}-\rho_C)y_j}{1-y_j}} - \sqrt{\frac{\rho_C z}{1-z}} < \frac{1}{\sqrt{\pi_j(1-\pi_j)}} - \sqrt{\frac{\rho_{CT}-\rho_C}{y_j(1-y_j)}} - \sqrt{\frac{\rho_C}{z(1-z)}} \tag{30}$$

$$\therefore \sqrt{\frac{\rho_{CT}-\rho_C}{y_j(1-y_j)}} - \sqrt{\frac{(\rho_{CT}-\rho_C)y_j}{1-y_j}} + \sqrt{\frac{\rho_C}{z(1-z)}} - \sqrt{\frac{\rho_C z}{1-z}} < \frac{1}{\sqrt{\pi_j(1-\pi_j)}} - \sqrt{\frac{\pi_j}{1-\pi_j}}$$

$$\therefore \sqrt{\frac{(\rho_{CT}-\rho_C)(1-y_j)^2}{y_j(1-y_j)}} + \sqrt{\frac{\rho_C(1-z)^2}{z(1-z)}} < \frac{1-\pi_j}{\sqrt{\pi_j(1-\pi_j)}}$$

$$\therefore \sqrt{\frac{(\rho_{CT}-\rho_C)(1-y_j)}{y_j}} + \sqrt{\frac{\rho_C(1-z)}{z}} < \sqrt{\frac{1-\pi_j}{\pi_j}}. \tag{31}$$

Also, again assuming that $m_j > 0$, $x_j > 0$ gives

$$\sqrt{\frac{\pi_j}{1-\pi_j}} - \sqrt{\frac{(\rho_{CT}-\rho_C)y_j}{1-y_j}} - \sqrt{\frac{\rho_C z}{1-z}} > 0 \tag{32}$$

$$\sqrt{\frac{(\rho_{CT}-\rho_C)y_j}{1-y_j}} + \sqrt{\frac{\rho_C z}{1-z}} < \sqrt{\frac{\pi_j}{1-\pi_j}}. \tag{33}$$

If we define $X'_j = 1 - X_j, Y'_j = 1 - Y_j, Z' = 1 - Z, W'_j = 1 - W_j$ then the W'_j will have the same correlation structure as the W_j but with complementary prevalences $\pi'_j = 1 - \pi_j$. In this case W'_j must also satisfy equivalent constraints, that is, the constraints must be symmetric when switching π_j, x_j, y_j, z for $1-\pi_j, 1-x_j, 1-y_j, 1-z$. We can see that Inequality (33) is just Inequality (31) with this replacement made, since it was derived from $x_j < 1 \Leftrightarrow 1 - x_j > 0$ rather than $x_j > 0$.

Further, if the inequalities (33) and (31) are satisfied and m_j are defined as in (27) then it will also be true that $m_j > 0$. This is because (33) is equivalent to (32) and (31) is equivalent to (30), and the expression on the left hand side of (30) is

the same as that on the left hand side of (32), and together they imply that

$$\frac{1}{\sqrt{\pi_j(1-\pi_j)}} - \sqrt{\frac{\rho_{CT}-\rho_C}{y_j(1-y_j)}} - \sqrt{\frac{\rho_C}{z(1-z)}} > 0 \tag{34}$$

$$\Leftrightarrow 1 - \sqrt{\frac{(\rho_{CT}-\rho_C)\pi_j(1-\pi_j)}{y_j(1-y_j)}} - \sqrt{\frac{\rho_C\pi_j(1-\pi_j)}{z(1-z)}} > 0 \tag{35}$$

$$\Leftrightarrow m_j > 0. \tag{36}$$

The remaining constraints of $0 < m_j, \widetilde{m}_j, z < 1$ will also be satisfied automatically when they are determined as in Equations (23), (26) and (27). Therefore, we need only satisfy Inequalities (31) and (33).

Let

$$q_{Y_j} = \sqrt{\frac{y_j}{1-y_j}}; \quad q_Z = \sqrt{\frac{z}{1-z}}; \quad q_{W_j} = \sqrt{\frac{\pi_j}{1-\pi_j}}, \tag{37}$$

the square-root of the odds ratios of Y_j, Z, and W_j respectively. The value of q_{W_j} is constrained by the problem, and we wish to find the values of q_{Y_j} and q_Z to simultaneously satisfy (31) and (33), that is,

$$\sqrt{\rho_{CT}-\rho_C}\,q_{Y_j} + \sqrt{\rho_C}\,q_Z < q_{W_j} \tag{38}$$

$$\frac{\sqrt{\rho_{CT}-\rho_C}}{q_{Y_j}} + \frac{\sqrt{\rho_C}}{q_Z} < \frac{1}{q_{W_j}} \tag{39}$$

Rewriting these as equalities allows us to find the boundaries of the feasible values for q_Z. If the feasible regions for (38) and (38) don't intersect then there will be no feasible solutions. Once we have chosen a value of q_Z we can then choose values of q_{Y_j} to satisfy the pair of inequalties (38), (39) for each j. Solving the equations

$$\sqrt{\rho_{CT}-\rho_C}\,q_{Y_j} + \sqrt{\rho_C}\,q_Z = q_{W_j} \tag{40}$$

$$\frac{\sqrt{\rho_{CT}-\rho_C}}{q_{Y_j}} + \frac{\sqrt{\rho_C}}{q_Z} = \frac{1}{q_{W_j}} \tag{41}$$

for one specific j gives

$$q_Z = \frac{q_{W_j}(1+2\rho_C-\rho_{CT}) \pm \sqrt{q_{W_j}^2(1+2\rho_C-\rho_{CT})^2 - 4\rho_C q_{W_j}^2}}{2\sqrt{\rho_C}}. \tag{42}$$

These two values of q_Z are the upper and lower bounds for the feasible values of q_Z for (38), (39) for that specific j. If the discriminant $q_{W_j}^2(1+2\rho_C-\rho_{CT})^2 - 4\rho_C q_{W_j}^2$ is less than 0 then there will be no feasible values of q_Z, this is equivalent to

$r = 2\sqrt{\rho_C}/(1 + 2\rho_C - \rho_{CT}) > 1$. Otherwise, let

$$q_{Z,L,j} = \frac{q_{W_j}(1 + 2\rho_C - \rho_{CT}) - \sqrt{q_{W_j}^2(1 + 2\rho_C - \rho_{CT})^2 - 4\rho_C q_{W_j}^2}}{2\sqrt{\rho_C}} \tag{43}$$

$$q_{Z,U,j} = \frac{q_{W_j}(1 + 2\rho_C - \rho_{CT}) + \sqrt{q_{W_j}^2(1 + 2\rho_C - \rho_{CT})^2 - 4\rho_C q_{W_j}^2}}{2\sqrt{\rho_C}}. \tag{44}$$

If there exists j_1, j_2 such that $q_{Z,L,j_1} > q_{Z,U,j_2}$ then there are no feasible solutions for q_Z. Otherwise, any value of q_Z in the interval $[q_{Z,L,j_1}, q_{Z,U,j_2}]$ will have a set of feasible q_{Y_j}. Pick a value q_Z^* such that $\max_j q_{Z,L,j} < q_Z^* < \min_j q_{Z,U,j}$; for example,

$$q_Z^* = \frac{\max_j q_{Z,L,j} + \min_j q_{Z,U,j}}{2}. \tag{45}$$

Then for each j choose $q_{Y_j} = q_{Y_j}^*$ to satisfy the pair of inequalities (38) and (39), *i.e.*

$$\frac{\sqrt{\rho_{CT} - \rho_C}}{1/q_{W_j} - \sqrt{\rho_C}/q_Z} < q_{Y_j} < \frac{q_{W_j} - \sqrt{\rho_C} q_Z}{\sqrt{\rho_{CT} - \rho_C}}. \tag{46}$$

In the special case where $\rho_{CT} = \rho_C$ the value on the right will be undefined, but in this case $\widetilde{m}_j = 0$ and the value of q_{Y_j} and y_j are irrelevant, and so any value can be chosen for y_j and simulation will remain feasible. Otherwise, one value of q_{Y_j} that satisfies (46) is

$$q_{Y_j}^* = \sqrt{\frac{q_{W_j}^* - \sqrt{\rho_C} q_Z}{1/q_{W_j}^* - \sqrt{\rho_C}/q_Z}}, \tag{47}$$

the geometric mean of the upper and lower bounds for q_{Y_j}.

This is now sufficient to determine y_j, z from the definitions (37) and $\overline{m}_j, \widetilde{m}_j, m_j$ from Equations (23), (26) and (27).
